# Expression and regulation of ATF6α in the mouse uterus during embryo implantation

**DOI:** 10.1186/s12958-016-0199-0

**Published:** 2016-10-07

**Authors:** Yongjie Xiong, Wenzhe Li, Pengfei Lin, Lei Wang, Nan Wang, Fenglei Chen, Xiao Li, Aihua Wang, Yaping Jin

**Affiliations:** 1 Key Laboratory of Animal Biotechnology of the Ministry of Agriculture, Northwest A&F University, Yangling, Shaanxi 712100 China; 2 College of Veterinary Medicine, Northwest A&F University, Yangling, Shaanxi 712100 China

**Keywords:** ATF6α, Progesterone, Estrogen, Uterus, Implantation, Decidualization

## Abstract

**Background:**

ATF6α, one of the sensor proteins in the stress signaling pathway of the endoplasmic reticulum, is located in the membrane of the endoplasmic reticulum. To date, the physiological function of ATF6α in the process of embryo implantation has not been reported.

**Methods:**

In this study, the expression pattern of ATF6α in the mouse uterus during peri-implantation and the estrous cycle was detected by real-time PCR, western blot and immunohistochemistry.

**Results:**

ATF6α mRNA and protein levels were higher in the uterus near the implantation site on day 5 and were intensely expressed in the secondary decidual zone (SDZ) on days 7–8. In the uteri of pseudopregnant mice, ATF6α mRNA and protein levels were lower on day 5 than on other days. The activating blastocyst and artificial decidualization had an obvious effect of increasing the expression of ATF6α. In addition, the expression of ATF6α was affected by progesterone (P_4_) and estrogen (E_2_) in ovariectomized mice. This finding is further supported by evidence from mice during the estrous cycle.

**Conclusions:**

Thus, we have concluded that ATF6α may play an important role during embryo implantation and decidualization.

**Electronic supplementary material:**

The online version of this article (doi:10.1186/s12958-016-0199-0) contains supplementary material, which is available to authorized users.

## Background

Embryo implantation is a complex process for the successful mammalian pregnancy and is affected by various factors [1–3]. The precise coordination between blastocyst activation and maternal endometrium receptivity is the most important step [4]. Once the apposition of the activated blastocyst is complete, the processes of adhesion and invasion must be initiated successively. A battery of physiological changes occurs in the cells and tissues of the uterus during early pregnancy, including morphological changes and the secretion of cytokines or proteins related to embryonic development and uterine receptivity [5, 6]. Implantation failure is one of the main causes of infertility. Although many molecular modulators have been identified as important for embryo implantation, the complete molecular mechanism is still unclear [7, 8].

Activating transcription factor 6 (ATF6) is a sensor protein in the membrane of the endoplasmic reticulum [9]. ATF6 includes two isoforms, known as ATF6α and ATF6β, which are members of the activating transcription factor (ATF)/cyclic adenosine monophosphate response element-binding protein (CREB) family [10]. Under the conditions of endoplasmic reticulum stress, the two isoforms are transported to the Golgi complex, where they are hydrolyzed into their cleaved forms, ATF6α (P50-ATF6α/N-ATF6α) and ATF6β (P50-ATF6β/N-ATF6β), by site-1 protease (S1P) and site-2 protease (S2P), respectively [11]. The cleaved ATF6 then translocates to the nucleus where it induces the expression of its downstream target genes [12, 13]. ATF6α plays a dominant role in inducing the expression of ATF6 target genes, while ATF6β plays a compensatory role in the absence of ATF6α [14–16].

In eukaryotes, the endoplasmic reticulum (ER) plays an important role in the synthesis and modification of proteins. The ER can maintain the self-homeostatic status by refolding or degrading misfolded or unfolded proteins [17]. Once the self-homeostatic status in the ER is disrupted by the overload of misfolded or unfolded proteins resulting from a variety of physiological or pathological factors, the ER stress response is initiated to reestablish self-homeostasis [18]. Previous studies have demonstrated that ER stress has an important effect on the reproductive processes in female mammals [19]. During embryo implantation, many genes involved in the ER stress response are up-regulated. For example, the expression level of Grp78, a marker of ER stress, is increased in the pregnant mouse uterus on day 5, and the positive immunoreactivity of Grp78 is present in the uterine luminal epithelia [20]; these results also imply that ER stress has an important effect on embryo implantation by regulating the expression of the ER stress response genes. The ATF6 protein, as a signal transducer, is essential in transmitting the stress signals during the ER stress response [21]. It has been proven that ATF6 plays various roles in a variety of physiological and pathological process in mammals. Deletion of ATF6α can result in an aggravation of Parkinson’s disease (PD) by accelerating neuronal degeneration and promoting ubiquitin accumulation [22]. The activation of ER stress signals mediated by ATF6 in mouse Leydig cells reduces the production of testosterone by regulating steroidogenic enzyme expression [23]. Moreover, a high level of cleaved ATF6 is predominantly maintained during the functional stage of the corpus luteum in mouse, implying that ATF6 is involved in sustaining the normal function of the corpus luteum [24]. The decreased expression of ATF6 in the placenta may be responsible for the pathophysiology of late-onset pre-eclampsia (PE) that is caused by the incomplete attachment of the blastocyst and the abnormal vascular remodeling of the placenta [25]. Additionally, several studies also have shown that ATF6 plays an important role in the female reproductive process; ATF6 has been detected in the embryonic extravillous trophoblast cells and contributes to blastocyst attachment, and knockout of both of the ATF6 isoforms causes embryonic lethality in mice [16, 25, 26]. To date, ATF6 and ER stress have been implicated in a variety of roles in the reproductive processes. However, an in-depth study of the expression and regulation of ATF6 in the mouse uterus during the early stages of pregnancy has not been performed. Furthermore, whether ATF6, as an important participant in the ER stress response, is involved in the successful implantation of the embryo remains largely elusive. Therefore, the aim of our study is to examine the expression and regulation of ATF6α in the mouse uterus during peri-implantation and to provide a basis for exploring the role of ATF6 in female reproduction. In this study, the main finding is that an increased level of ATF6α is present in the uterus with an implantation site on pregnancy day 5, a crucial stage of embryo implantation, indicating that up-regulation of ATF6α may have a close association with the success of embryo implantation.

## Methods

### Animals and treatments

Mature male and female mice (Kunming White outbred strain) were purchased from the laboratory animal center of Xi’An JiaoTong University. The mice were housed in a 25 ± 1 °C environmentally controlled and artificially lighted room (12 h of light: 12 h of darkness) with food and water provided ad libitum. All procedures were approved by the Committee for the Ethics on Animal Care and Experiments of Northwest A&F University.

We established the experimental mouse models of normal pregnancy at days 1–8, pseudopregnancy at days 1–5, stromal-cell induced decidualization, delayed and activation-implantation, and steroid hormone regulation using previously described procedures [27, 28]. The mouse models of normal pregnancy were prepared by following methods. In brief, adult female and male mice were mated at 5:00 PM. At 9:00 AM on the morning of the second day, those mice presenting with a vaginal plug were considered as pregnant day 1. The pregnant mice from day 1 to 4 were further confirmed by flushing the oviducts and uteri to confirm the presence of an embryo. For the pregnant mice on days 5–8, trypan blue (0.2 ml/mouse, 1 % in normal saline; Sigma-Aldrich Corp., St. Louis, MO, USA) was injected intravenously before the mice were euthanized to identify the implantation site. The pregnant mice on days 1–8 were sacrificed to collect the uteri at 9:00 AM for further analysis.

Vasectomized and proven sterile male mice were used to produce pseudopregnant mice. Using a similar approach, the female mice presenting with a vaginal plug were considered as pseudopregnant day 1. The uteri were collected from pseudopregnant mice on days 1–5. For the pseudopregnant mice on day 4, one of the uterine horns was infused with 30 μl sesame oil to induce artificial decidualization, while the non-injected contralateral uterine horn served as the non-decidualization control. The uteri that were injected and non-injected sesame oil were collected on day 8 of pseudopregnancy. The induced decidualization was confirmed by weighing the uterine horn and through the histomorphological examination of the uterine sections.

The delayed implantation model was made by ovariectomizing the pregnant mice on day 4 at 8:00–9:00 AM under anesthesia, followed by subcutaneous injection of progesterone (P_4_, 1 mg/mouse/day; Sigma-Aldrich Corp., St. Louis, MO, USA) on days 5–7. Implantation was subsequently activated by injecting estradiol (E_2_, 25 ng/mouse, Sigma-Aldrich Corp., St. Louis, MO, USA), and the uteri were collected 24 h later. Flushing of the blastocyst from one uterine horn of each uterus was performed to confirm the delayed implantation.

In the steroid hormone treatment experiment (fulvestrant and mifepristone; Sigma-Aldrich Corp., St. Louis, MO, USA), the female mice were ovariectomized before 2 weeks. Sesame oil was used as the solvent to prepare the hormone solutions. The ovariectomized mice were injected subcutaneously with either E_2_ (100 ng/mouse), P_4_ (1 mg/mouse), E_2_ (100 ng/mouse) plus P_4_ (1 mg P_4_/mouse), E_2_ (100 ng/mouse) plus fulvestrant (1000 ng/mouse), or P_4_ (1 mg P_4_/mouse) plus mifepristone (10 mg/mouse). Sesame oil alone was injected into the control group mice. After treatment for 24 h, the uterine tissues from all the treated mice were collected for further analysis. The phases of the estrous cycle were tracked daily and were confirmed based on vaginal cell smears. Briefly, a cotton swab wetted in normal saline with the same ambient temperature was gently inserted into the vagina of the mouse and was rolled. Then, the swab was removed and was wiped on a dry glass slide. By this method, cells were transferred and adhered to the glass slide. Subsequently, the glass slide was treated using a standard procedure of hematoxylin-eosin staining, and the slide was viewed under a digital microscope (BA400, Motic, Wetzlar, Germany). Both nucleated and cornified epithelial cells were prevalent on the glass slide from female mice in proestrus. When the female mice were in estrus, only the cornified epithelial cells were observed. If both cornified epithelial cells and leukocytes were observed, this indicated that the female mouse was in metestrus. However, in late metestrus, a few nucleated epithelial cells could also be observed. The leukocytes were predominant with a few nucleated epithelial cells during diestrus. Only those mice that exhibited regular 4-day or 5-day estrous cycles were subjected to uterine horn collection in the process of the estrous cycle. Each uterus subjected to the same treatment was cut into three pieces following sacrifice for the extraction of RNA and proteins and immunohistochemistry. In this study, a total of 265 mice were used in all of the experiments. The analysis of each sample was performed in triplicate.

### Immunohistochemical staining

Immunohistochemistry was performed as previously described [29]. Uterine tissue was immersed in a 4 % formaldehyde solution for 24 h. Then, the tissue was dehydrated gradually in serial concentrations of alcohol and was completely immersed in liquid paraffin. Once solidified, the paraffin was cut into sections at a thickness of 6 μm. The sections were adhered to the surface of glass slides treated with polylysine (Sigma-Aldrich Corp., St. Louis, MO, USA) and were maintained in an incubator at 37 °C for 12 h. The incubated sections were successively immersed in a series of alcohol solutions at different concentrations to remove the paraffin adhered to the sections. Antigen retrieval was carried out by submerging the slices in a citric acid salt mixture (100 mM citrate and 100 mM Na-citrate) for 15 min at 90 °C with a microwave oven. The samples were cooled to room temperature and added to a solution consisting of hydrogen peroxide and methyl alcohol (3 % *v/v*) for 15 min, followed by three washes in phosphate buffer. The slices were then treated using an immunohistochemical staining kit (Maixin Biotech. Co., Ltd. Fuzhou, China). Briefly, the samples were first blocked using 10 % goat serum (reagent B in the kit) for 1 h at 37 °C. Then, the samples were incubated with rabbit anti-ATF6α antibody (SC-22799, 1:100, Santa Cruz Biotechnology, Inc., Dallas, Texas, USA) at 4 °C overnight and washed three times with phosphate buffer. The sections were then incubated with goat anti-rabbit antibody (reagent C in the kit) for 1 h at 37 °C and washed three times again with phosphate buffer. The slides were incubated with horseradish peroxidase (HRP)-streptavidin (reagent D in the kit) for 20 min at 37 °C. Finally, the sections were treated with 3, 3′-diaminobenzidine (DAB) (Sigma-Aldrich Corp., St. Louis, MO, USA) and hematoxylin. For the sections from the negative control group, the ATF6α antibody was replaced with normal rabbit IgG (SC-2763, 1:100, Santa Cruz Biotechnology, Inc., Dallas, Texas, USA) according to our previous report [29].

### Total RNA extraction and cDNA synthesis

Following the manufacturer’s instructions, total RNA was extracted from the mouse uterine tissue by treatment with TRIzol and deoxyribonuclease (Takara Biotechnology, Co., Ltd., Dalian, China). The total RNA concentration was measured as previously described [29]. cDNA was synthesized using a reverse transcription kit (Takara Biotechnology, Co., Ltd., Dalian, China). Extracted total RNA samples and the synthesized cDNAs were stored at −80 °C.

### Real-time PCR

The following primer sequences were used for real-time PCR: ATF6α (NM_001081304.1), forward-primer, 5′-TCGCCTTTTAGTCCGGTTCTT, reverse-primer, 5′-GGCTCCATAGGTCTGACTCC; GAPDH (NM_008084.3), forward-primer, 5′-TCACTGCCACCCAGAAGA, reverse-primer, 5′-GACGGACACATTGGGGGTAG; RPLP0 (NM_007475.5), forward-primer, 5′-GGACCCGAGAAGACCTCCTT, reverse-primer, 5′- GCACATCACTCAGAATTTCAATGG. Real-time PCR was performed using SYBR® Premix Ex Taq™ (Perfect Real Time) (Takara Biotechnology, Co., Ltd., Dalian, China), and the data were analyzed using the Bio-Rad iQ5 system (Bio-Rad Laboratories, Inc., Hercules, California, USA) following the manufacturer’s instructions. Each PCR reaction contained the following reagents: 2.0 μl reverse transcription products (containing 20 ng cDNA), 1.6 μl primer mixture, 10.0 μl SYBR Premix Ex TaqTM II and 6.4 μl ribonuclease-free water. The PCR cycling conditions were as follows: denaturation at 95 °C for 30 s, followed by 40 cycles at 95 °C for 5 s and 60 °C for 20 s. To ascertain the specificity of the PCR amplification, a melting curve analysis was performed including the negative control group (no template cDNA). The relative mRNA expression levels were determined using the 2^-△△Ct^ method; GAPDH and RPLP0 were used as the internal reference genes and the geometric averaging of these two reference genes were used to normalize the relative mRNA expression according to previous reports [30–32].

### Western blotting

The extraction, concentration, and detection of proteins from uterine tissues were completed using a protein extraction and examination kit (Jiangsu Keygen Biological Technology Co., Ltd, Nanjing, China), according to the manufacturer’s instructions. Protein samples (30 μg) were resolved by electrophoresis on a 12 % sodium dodecyl sulfate-polyacrylamide gel. After electrophoresis, the resolved proteins were transferred to a polyvinylidene fluoride membrane (Millipore, Co. Ltd., Shanghai, China). The membrane was then blocked by immersion in Tris-buffered saline (TBS) containing 10 % nonfat-dried milk for 60 min at room temperature (RT). Then, the membranes were incubated with anti-ATF6α antibody (1:200, SC-22799; Santa Cruz Biotechnology, Inc., Dallas, Texas, USA) or β-actin as a loading control (CW0096, 1:1000, CWBIO Biological Technology Co. Ltd. Beijing, China) for 12 h at 4 °C. After washing with TBS, the membranes were incubated with horseradish peroxidase conjugated anti-rabbit IgG antibody (1:6000; SUNGENE Biotech Co. Ltd., Tianjin, China) at RT for 60 min. After washing, the immunoblotting signals were analyzed using the Gel Photograph System (Tanon Technology Co. Ltd., Shanghai, China). According to the manufacturer’s instructions, western blot analysis of the cleaved ATF6α expression in rat liver extract (SC-2395, 1:200, Santa Cruz Biotechnology, Inc., Dallas, Texas, USA) was performed as a positive control. The normal rabbit IgG was used as the negative non-relevant IgG control.

### Data statistics and analysis

Throughout this study, all experiments were replicated at least three times for each group or each treatment (*n* ≥ 3). The data are presented as the mean ± S.E.M. ANOVA, Fisher LSD and independent sample T-tests were conducted using SPSS statistical software (version 19.0, Chicago, IL, USA). Differences were considered significant if *P* < 0.05.

## Results

### ATF6α expression in the uteri of pregnant mice during days 1–8

**Fig. 1:**
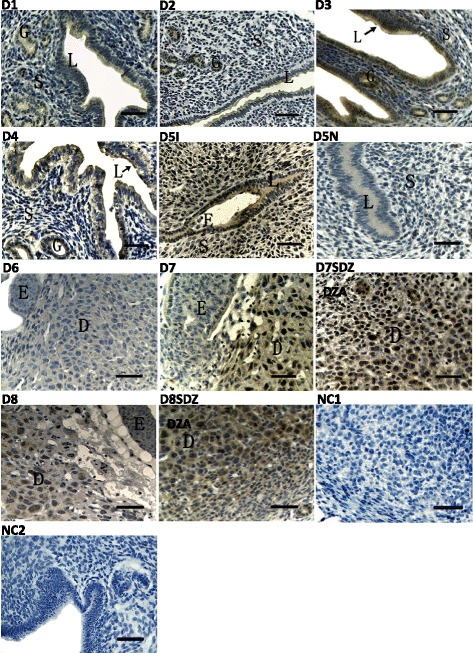
Immunohistochemical staining of ATF6α protein in the mouse uterus during pregnancy from days 1 to 8 (*n* ≥ 3). D1-D4, uterine sections from days 1 to 4 of pregnancy; D5I, implantation sites on day 5 of pregnancy; D5N, non-implantation sites on day 5 of pregnancy; D6, the primary decidual zone (PDZ) in pregnancy day 6; D7, the primary decidual zone (PDZ) in pregnancy day 7; D7SDZ, the secondary decidual zone (SDZ) in pregnancy day 7; D8, the primary decidual zone (PDZ) in pregnancy day 8; D8SDZ, the secondary decidual zone (SDZ) in pregnancy day 8; NC1, negative control including decidua cells; NC2, negative control including luminal epithelium and glandular epithelium. L luminal epithelium, G glandular epithelium, S stromal cells, D decidual cells, E embryo. Scale bar 40 μm

**Fig. 2:**
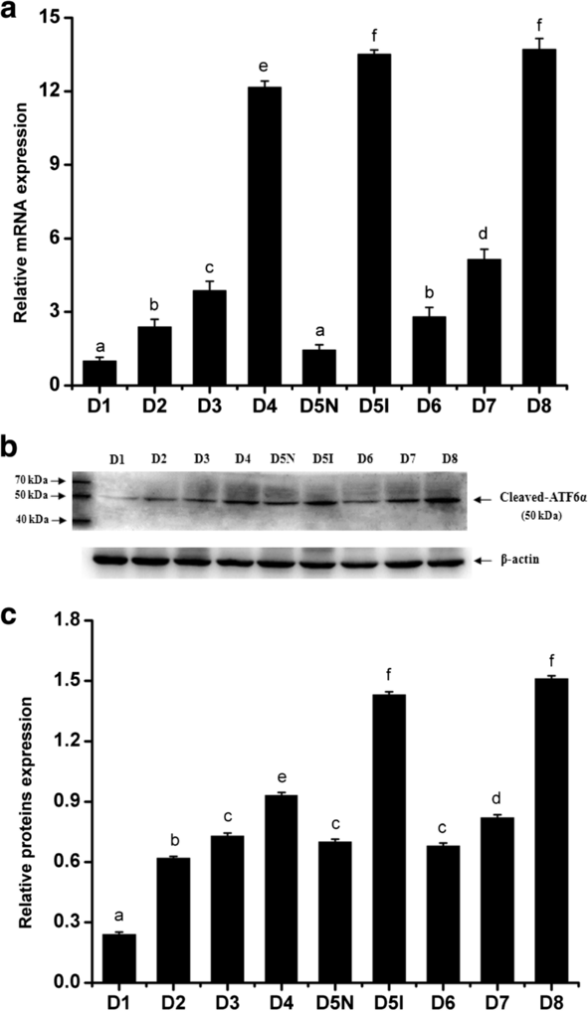
The expression levels of ATF6α mRNA and protein in the mouse uterus during pregnancy were detected by RT-PCR (**a**) and western blotting (**b** and **c**), respectively (*n* ≥ 3). D1–D8, uterine sections from days 1 to 8 of pregnancy; D5N, non-implantation sites on day 5 of pregnancy; D5I, implantation sites on day 5 of pregnancy. The results of statistical analysis are shown as histograms. Different lowercase letters on the bars indicate significant differences between specific two groups (‘a’ and ‘a’, ‘b’ and ‘b’, ‘f’ and ‘f’, etc., *P* < 0.05; ‘a’ and ‘b’, ‘b’ and ‘c’, ‘c’ and ‘e’, etc., *P* < 0.05 or *P* < 0.01)

To elucidate the spatial and temporal distribution of ATF6α in the mouse uterus early in pregnancy, we analyzed the expression pattern of ATF6α in mouse uteri during peri-implantation by immunohistochemical staining, real-time PCR, and western blot. It was found that ATF6α proteins were mainly localized in the endometrium luminal cells and glandular epithelial cells during days 1 to 4 of pregnancy (Fig. 1, D1–D4). Moreover, the expression levels of ATF6α mRNA and protein were significantly increased from day 1 to day 5, with the highest levels detected in the pregnant uterus on day 5 within the implantation site (*P* < 0.01, Fig. 2a–c). Notably, on pregnancy day 5, a large number of endometrium luminal, glandular epithelial and stromal cells with immunostaining of ATF6α were present in the uterus with an implantation site, whereas the positive immunostaining of ATF6α was hardly observed in these cells in uteri without an implantation site (Fig. 1, D5I–D5N). Additionally, as decidualization proceeded during days 6–8, immunohistochemistry demonstrated that the ATF6α protein was present in the primary decidualization zone (PDZ) and the secondary decidualization zone (SDZ) (Fig. 1, D6-D8SDZ). Simultaneously, although the results from real-time PCR and western blot indicated that the expression levels of ATF6α mRNA and protein were relatively low at the beginning of decidualization on day 6, the levels of ATF6α mRNA and protein were progressively increased over the whole process of decidualization from days 6–8 (*P* < 0.05 or *P* < 0.01, Fig. 2a–c). Western blot analysis of cleaved ATF6α expression in rat liver extract was performed as a positive control. The normal rabbit IgG was used as the negative non-relevant IgG control. The results shown are representative of 3 independent experiments (Additional file 1).

### ATF6α expression in pseudopregnant uteri during days 1–5

**Fig. 3:**
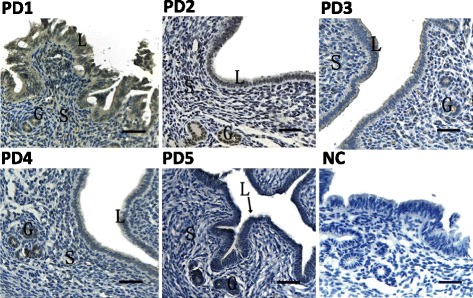
Immunohistochemical staining of ATF6α protein in the uterus of pseudopregnant mice (*n* ≥ 3). PD1–PD5, uterine sections from days 1–5 of pseudopregnancy; NC, negative control. L luminal epithelium, G glandular epithelium, S stromal cells, Scale bar 40 μm

**Fig. 4:**
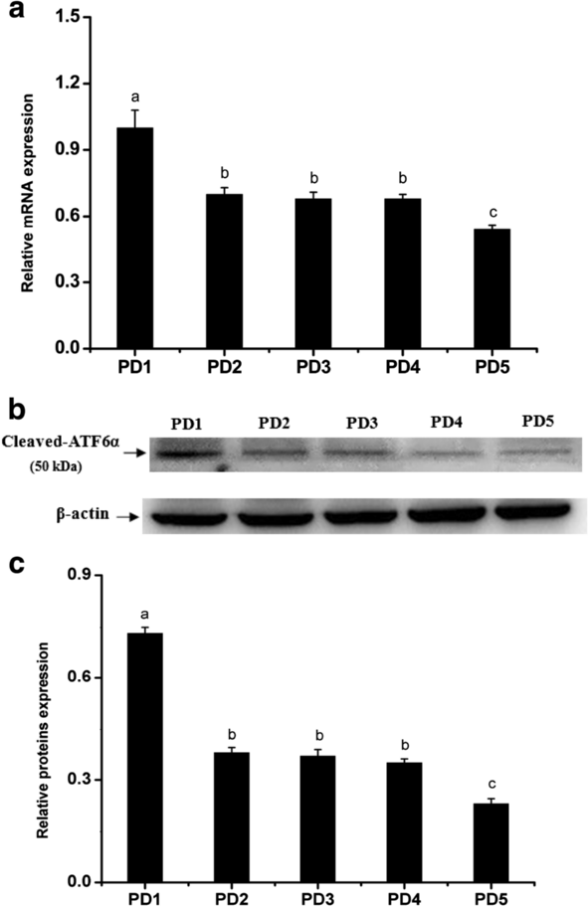
The expression levels of ATF6α mRNA and protein in the mouse uterus of pseudopregnancy detected by RT-PCR (**a**) and western blotting (**b** and **c**), respectively (*n* ≥ 3). PD1-PD5, uterine sections from days 1 to 5 of pseudopregnancy. The results of statistical analysis are shown as histograms. Different lowercase letters on the bars indicate significant differences between specific two groups (‘a’ and ‘b’, ‘b’ and ‘c’, *P* < 0.05 or *P* < 0.01; ‘b’ and ‘b’, *P* > 0.05)

To assess the effect of the blastocyst on ATF6α expression in the pregnant mouse uterus, a pseudopregnancy model was used. In the pseudopregnant mouse uterus, the ATF6α protein was mainly distributed in the glandular and luminal epithelial cells on days 1–4, while the immunostaining was barely present on day 5 (Fig. 3). Moreover, the real-time PCR and western blot results demonstrated that the expression levels of ATF6α mRNA and protein were significantly decreased in the pseudopregnant uteri from days 1–2, and the levels of ATF6α mRNA and protein were down-regulated again from days 4–5 (*P* < 0.05 or *P* < 0.01, Fig. 4).

### ATF6α expression in delayed implantation and artificial decidualization

**Fig. 5:**
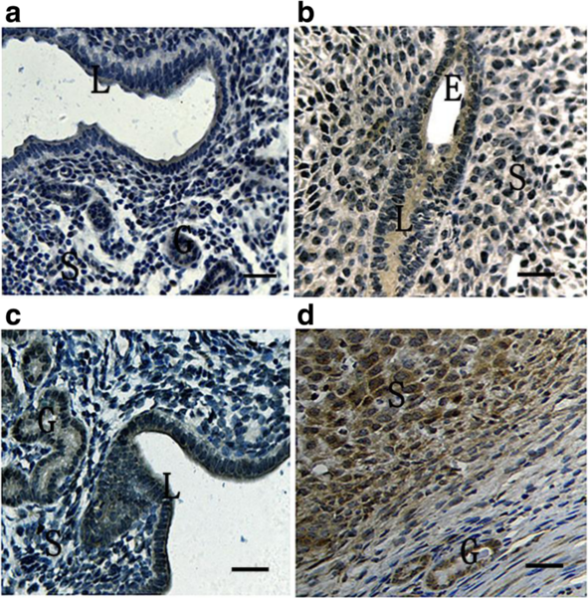
Immunohistochemical staining of ATF6α protein in the mouse uterus under delayed and activated implantation and decidualization (*n* ≥ 3). **a** delayed-implantation uteri; **b** activated-implantation uteri; **c** non-decidualization (control groups); **d** artificial decidualization uteri. L luminal epithelium, G glandular epithelium, S stromal cells, D decidual cells, E embryo. Scale bar 40 μm

**Fig. 6:**
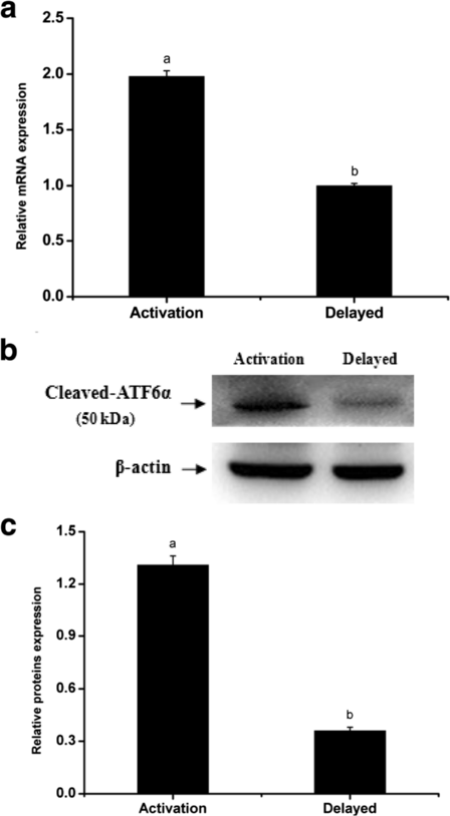
The expression levels of ATF6α mRNA and protein in the mouse uterus under delayed implantation and activated implantation were detected by RT-PCR (**a**) and western blotting (**b** and **c**), respectively (*n* ≥ 3). The results of statistical analysis are shown as histograms. Different lowercase letters on the bars indicate significant differences between specific two groups (‘a’ and ‘b’, *P* < 0.01)

**Fig. 7:**
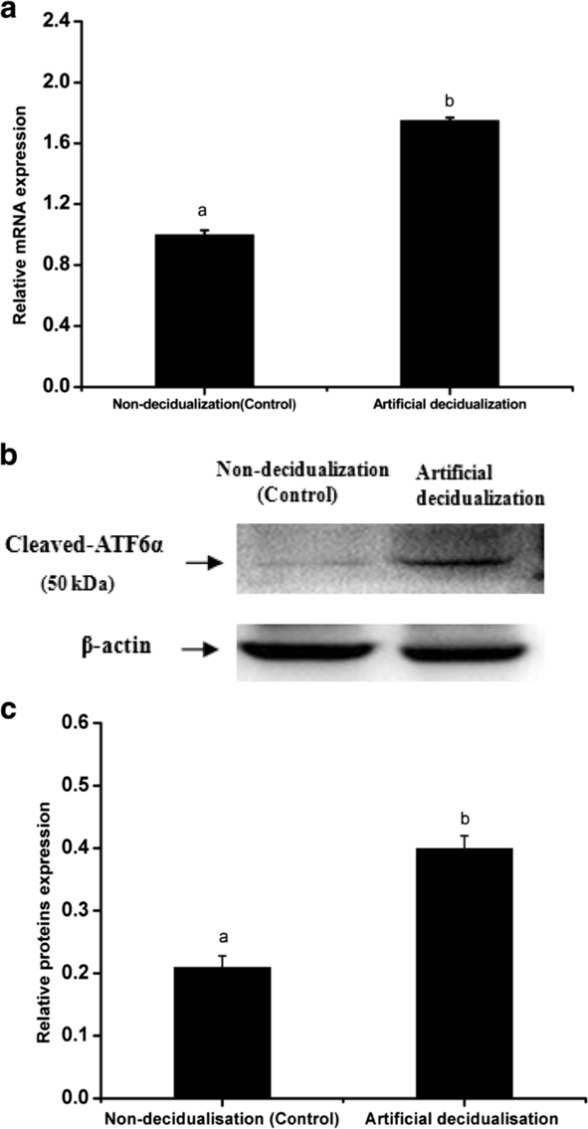
The expression levels of ATF6α mRNA and protein in the mouse uterus under artificial decidualization were detected by RT-PCR (**a**) and western blotting (**b** and **c**), respectively (*n* ≥ 3). The results of statistical analysis are shown as histograms. Different lowercase letters on the bars indicate significant differences between specific two groups (‘a’ and ‘b’, *P* < 0.01)

The delayed implantation model was utilized to further investigate whether the existence of active embryos is essential for ATF6α expression, and the artificial decidualization model was utilized to elucidate whether ATF6α is involved in the process of the decidualization. Compared with the delayed implantation uteri, although the ATF6α immunohistochemical staining was also present in the luminal epithelial cells and stromal cells of activated-implantation uteri (Fig. 5a–b), the expression levels of ATF6α mRNA and protein were higher in activated-implantation uteri than in delayed-implantation uteri (*P* < 0.01, Fig. 6a–c). Under artificial decidualization, positive staining of the ATF6α protein was located in the decidual cells. In the non-decidualization uterus, ATF6α protein expression was distributed in the luminal epithelia and glandular epithelia (Fig. 5c–d). In addition, significantly higher expression levels of ATF6α mRNA and protein were detected in the uterine horn injected with oil to induce artificial decidualization than in the control non-injected uterine horn (*P* < 0.01, Fig. 7a–c).

### Regulation of ATF6α expression in the mouse uterus via steroid hormones and antagonists

**Fig. 8:**
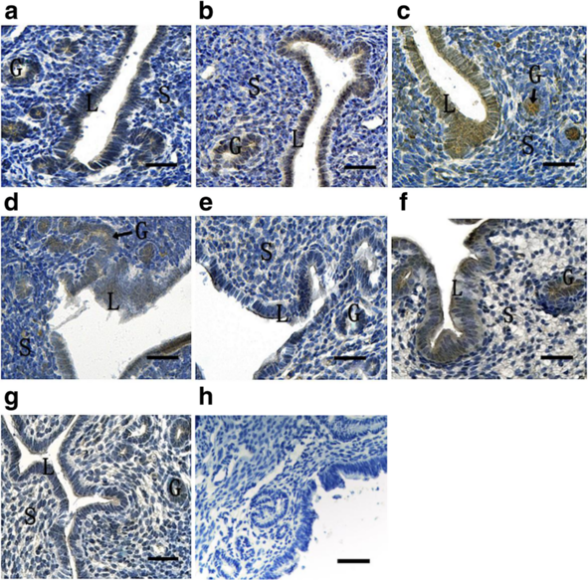
Immunohistochemical staining of ATF6α protein in the uterus of ovariectomized mice (*n* ≥ 3). **a** Control group; **b** E_2_-treated group; **c** P_4_ treated group; **d** E_2_ and P_4_ treated group; **e** P_4_ plus mifepristone-treated group; **f** E_2_ plus fulvestrant-treated group; **g** co-treatment of E_2_, P_4_, mifepristone and fulvestrant; **h** negative control group. L luminal epithelium, G glandular epithelium, S stromal cells. Scale bar 40 μm

**Fig. 9:**
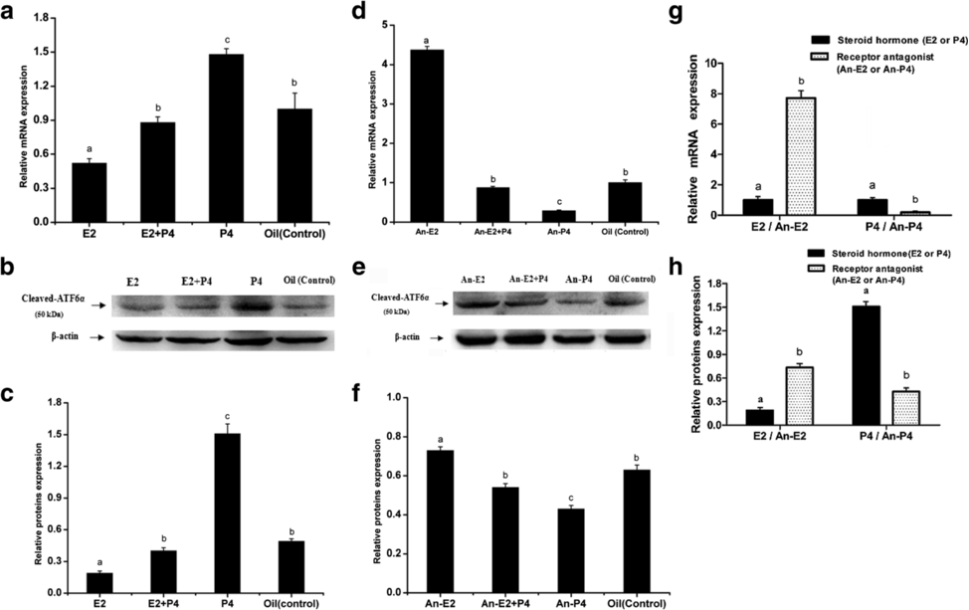
The expression levels of ATF6α mRNA and protein in the uterus of ovariectomized mice detected by Real-time PCR (**a**, **d** and **g**), western blotting (**b**, **c**, **e**, **f** and **h**), respectively (*n* ≥ 3). The results of statistical analysis are shown as histograms. E2 the E_2_-treated group, E2 + P4 the E_2_ plus P_4_ treated group, P4 the P_4_-treated group, Oil the control group, An-E2 fulvestrant plus E_2_ treated group, An-E2 + P4 co-treatment of mifepristone, fulvestrant, E_2_ and P_4,_An-P4 mifepristone plus P_4_ treated group. Different lowercase letters on the bars indicate significant differences between specific two groups (‘a’ and ‘b’, ‘b’ and ‘c’, ‘a’ and ‘c’, etc., *P* < 0.05 or *P* < 0.01; ‘b’ and ‘b’, *P* > 0.05)

To determine the effect of hormones on the expression of ATF6α in the mouse uterus, we performed the steroid hormone treatments in the ovariectomized mice. The result of immunohistochemistry showed that the ATF6α protein was mainly distributed in the uterine glandular and luminal epithelial cells while only a few stromal cells with ATF6α immunostaining were observed in the mouse uteri (Fig. 8). Compared with the control mice injected with oil only, the ATF6α mRNA and protein levels were both significantly increased following treatment with P_4_ (*P* < 0.05, Fig. 9a–c). Compared with the P_4_-treatment group, the ATF6α mRNA and protein levels were significantly reduced after treatment with E_2_ (*P* < 0.05, Fig. 9a–c). Furthermore, compared with the control mice, the ovariectomized mice treated with a combination of P_4_ and mifepristone (or RU-486, a P_4_ receptor antagonist) showed significantly down-regulated ATF6α mRNA and protein levels in their uteri (*P* < 0.05, Fig. 9d–f), while the expression of ATF6α mRNA and protein was significantly up-regulated after treatment with a combination of E_2_ and fulvestrant (an E_2_ receptor antagonist) (*P* < 0.05, Fig. 9d–f). Notably, compared to the down-regulation of the ATF6α mRNA and protein levels in the E_2_-treatment group, the ATF6α levels were up-regulated in the group treated with a combination of fulvestrant and E_2_ (*P* < 0.01, Fig. 9g–h). The ATF6α mRNA and protein levels were increased in the P_4_-treatment group whereas the levels were decreased in the group treated with a combination of mifepristone and P_4_ (*P* < 0.01, Fig. 9g–h).

### ATF6α expression in the mouse uterus during the estrous cycle

**Fig. 10:**
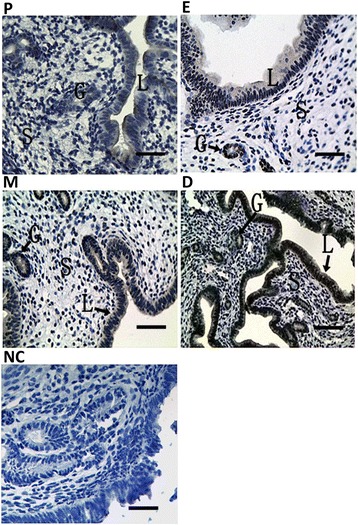
Immunohistochemical staining of ATF6α protein in the mouse uterus during the estrous cycle (*n* ≥ 3). (*P*) proestrus; (*E*) estrus; (*M*) metestrus; (*D*) dioestrous; (*NC*) negative control. L luminal epithelium, G glandular epithelium, S stromal cells. Scale bar 40 μm

**Fig. 11:**
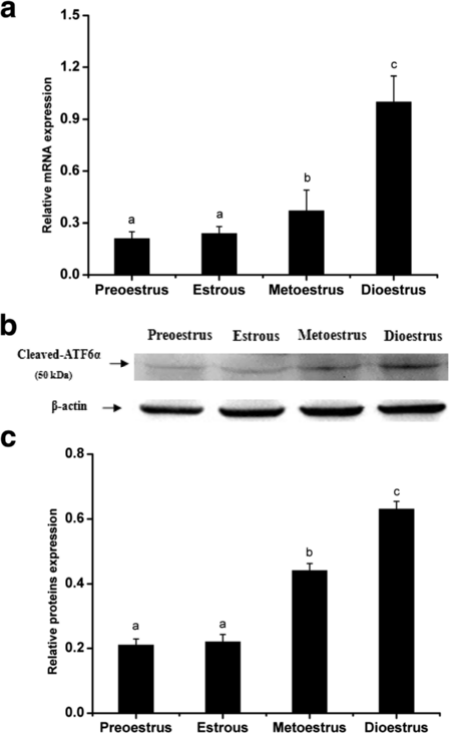
The expression levels of ATF6α mRNA and protein in the mouse uterus during the estrous cycle detected by RT-PCR (**a**) and western blotting (**b** and **c**), respectively(*n* ≥ 3). The results of statistical analysis are shown as histograms. Different lowercase letters on the bars indicate significant differences between specific two groups (‘a’ and ‘b’, ‘b’ and ‘c’, *P* < 0.05 or *P* < 0.01; ‘a’ and ‘a’, *P* > 0.05)

To further elucidate the relationship between hormone levels and ATF6α expression in the uterus, the ATF6α expression pattern was investigated during the mouse estrous cycle. Throughout the estrous cycle, positive ATF6α immunostaining was localized to the glandular and luminal epithelia, while only trace levels of ATF6α was observed in the stromal cells (Fig. 10). Moreover, the ATF6α mRNA level was lower in the proestrous and estrous phases than in all other phases, but increased gradually from the metestrous to dioestrous phases (*P* < 0.05 or *P* < 0.01, Fig. 11a). In line with the real-time PCR results, western blotting suggested that the ATF6α protein expression were gradually increased from the metestrous to dioestrous phases and the highest protein level was observed during the dioestrous phase (*P* < 0.05, Fig. 11b–c).

## Discussion

The role of ATF6 in the embryo implantation process of female mammals has not been obviously reported, particularly its expression and regulation in regards to the uterus. This research depicts for the first time the spatiotemporal expression pattern of ATF6α, an isoform that plays a prominent role in the activated form of ATF6, throughout the uterus during the early stages of pregnancy and all stages of the estrous cycle in the mouse.

It is well known that the pregnancy stage during days 1–5 is critical for successful embryo implantation, and the uterus is subjected to a variety of morphological and physiological function changes during this phase. Blastocyst attachment and invasion that occur on pregnancy day 5 are key steps for a successful pregnancy. During days 4 to 5 of pregnancy, the stage usually referred to as the implantation window, the blastocyst migrates from the fallopian tube and enters the uterus to complete the implantation process that includes blastocyst apposition, attachment and invasion [33, 34]. Moreover, previous studies have documented that the increased protein synthesis in the uterus of early pregnancy is closely associated with the uterus receptivity and blastocyst attachment [35, 36]. In the current study, ATF6α mRNA and protein levels were found to incrementally increase in the pregnant uterus from days 1–5 and the ATF6α immunostaining was mainly localized to the luminal and glandular epithelia, suggesting that ATF6α may be involved in the changes in morphology and physiological function resulting from the proliferation and differentiation of the luminal epithelium and glandular epithelium cells in the early pregnant uterus. Most notably, after implantation occurred on day 5, a large number of uterine luminal epithelial cells and stromal cells expressing ATF6α were observed in the uterus near the implantation site compared to the uterus without an embryo on day 5, indicating that ATF6α is closely related to the establishment of the implantation window and the process of embryo implantation. In the meantime, real-time PCR and western blotting showed that the ATF6α expression was significantly higher in a uterus with an implantation site than in a uterus without an implantation site. Moreover, a prior study demonstrated that ATF6α is abundant in the extravillous trophoblasts that are important participants in blastocyst invasion [26]. Hence, it may be inferred that ATF6α participates in the attachment and invasion of the blastocyst, or it may be an important regulator involved in the interaction between the blastocyst and the uterine epithelia during peri-implantation. In addition, it has also documented that the cellular processes of blastocyst trophoblast invasion and in cancer cell invasion are similar [37]. ATF6α expression levels correlated with the proliferation or apoptosis of cancer cells and the increased ATF6α levels promote the growth and proliferation of tumor cells [38], including ovarian cancer and endometrial adenocarcinoma cells [39, 40]. Taken together, we speculate that the up-regulation of ATF6α in the uterus with an implantation site on pregnancy day 5 may function to promote the invasion of the blastocyst trophoblasts into the uterine epithelia.

In the present study, the immunostaining staining of ATF6α protein was mainly distributed in the luminal epithelia, glandular epithelia and stromal cells during pseudopregnancy days 1–4. Moreover, the ATF6α immunoreactivity was hardly observed in the pseudopregnant uterus on day 5, demonstrating that ATF6α might be closely associated with blastocyst attachment. Meanwhile, examination of pseudopregnant mice revealed that the ATF6α mRNA and protein levels were significantly decreased from days 1–2,suggesting that ATF6α expression might be affected by the presence of blastocyst. In particular, ATF6α mRNA and protein levels significantly decreased even further from days 4–5, a key stage of embryo implantation, and the expression of ATF6α was lower in the uterus on day 5 than on all other days, further indicating that the existence of an embryo affects the level of ATF6α in the mouse uterus and significantly induces its expression during embryo implantation. This inference is further supported by the results of delayed-implantation and activated-implantation. Although ATF6α was present in both the uterine luminal epithelium and stromal cells in the uteri of delayed-implantation and activated-implantation, real-time PCR and western blotting showed that the level of ATF6α mRNA and protein was higher in the activated-implantation. Moreover, this ATF6α expression pattern in the activated-implantation uterus was similar to the expression pattern observed in the uterus with an implantation site on normal pregnancy day 5. The similarity of these results also support the inferences above that the expression of ATF6α is affected by the presence of a blastocyst and is induced by the implanted embryo. Additionally, this result might also be considered another powerful example of the activated embryo secreting some functional factors that can regulate the genes expressed in the uterus to affect the receptivity of the uterus and to ensure embryo implantation [41].

To complete successful implantation, the endometrial stromal cells near the implantation site begin to proliferate and then differentiate into decidualization cells during pregnancy days 6–8 [42]. A vascular connection between the embryo and the maternal uterus is established in coordination with the decidualization, which is a key step for successful embryo implantation [43]. Importantly, the impairment of decidualization results in embryo implantation failure and abortion [44]. The present study found that the ATF6α was present in the stromal cells of the pregnant uterus with an implantation site on day 5 whereas ATF6α expression was negligible in the stromal cells from the pregnant uterus without an implantation site. Moreover, the level of ATF6α was higher in the pregnant uterus with an implantation site on day 5. These results indicate that the implanted embryo might stimulate ATF6α expression, and ATF6α may be involved in the initial stages of decidual cell cytopoiesis from the proliferation and differentiation of stromal cells. During the process of stromal cell decidualization from pregnancy days 6–8, ATF6α was present in the primary decidual zone (PDZ) and secondary decidual zone (SDZ), and the levels of both ATF6α mRNA and protein gradually accumulated during days 6–8. This up-regulation of ATF6α is in line with the increase in decidual cells through the proliferation and differentiation of stromal cells, indicating that ATF6α may be an important participant or modulator in the process of decidualization. Interestingly, the ATF6α expression level was lower in the uterus when decidualization began on pregnancy day 6, but it was higher in the uterus containing the secondary decidual zone (SDZ) when decidualization was occurring at a later stage of pregnancy (day 8). Taken together, these results imply that ATF6α may play an important role in the process of decidualization, particularly in the later stages on days 7–8, during which the endometrial cells differentiate into multiploid decidual cells. In fact, this implication was further supported by the higher levels of ATF6α expression in the decidual uterus upon induction of artificial decidualization.

Previous reports have shown that estrogen (E_2_) and progesterone (P_4_) are indispensable for successful embryo implantation and normal endometrial cell decidualization [45, 46]. Our research revealed that P_4_ could markedly promote the expression of ATF6α in the uterus of ovariectomized mice, while E_2_ inhibited the expression of ATF6α. These results were also confirmed by the expression pattern of ATF6α in the mice treated with a combination of P_4_ and an antagonist of the P_4_ receptor, or a combination of E_2_ and an antagonist of the E_2_ receptor. Mifepristone, an antagonist of the P_4_ receptor, suppressed the expression of ATF6α in the uterus of ovariectomized mouse while fulvestrant, an antagonist of the E_2_ receptor, induced the expression of ATF6α. This relationship between ATF6α and hormone levels was further supported by the findings from mice during the estrous cycle. Throughout the estrous cycle of the mouse, a relatively higher level of E_2_ and lower level of P_4_ are present in estrus, a stage in which the female mouse ovulates and has optimal sexual receptivity, but the E_2_ level decreases gradually while the P_4_ level increases from metestrus to diestrus [47]. We found that ATF6α expression increased gradually in the mouse uterus from metestrus to diestrus. This change is coincident with the change in the steroid hormone levels from metestrus to diestrus. In short, all of these results suggest that P_4_ has a promoting effect on ATF6α expression and E_2_ has an antagonistic effect on P_4_-dependent up-regulation of ATF6α. ATF6α might enhance or inhibit the function of steroid hormones during the processes of implantation and successful decidualization due to the regulatory actions of different steroid hormones.

Based on the current results, it was found that the distribution of ATF6α in the mouse uterus was diverse, and its expression was affected by a variety of factors. In particular, both the activated blastocyst and hormones have important effects on the level of ATF6α in the mouse uterus during early pregnancy. In a normal early pregnancy mouse uterus, the higher level of E_2_ on pregnancy day 1 stimulates the growth of the uterine epithelial cells and promotes the induction of P_4_ receptors. However, the level of E_2_ is decreased from pregnancy days 2–3. Subsequently, the endometrial receptivity is induced by the combination of a surge of E_2_ and a high level of P_4_ on pregnancy day 4 to accommodate the forthcoming blastocyst attachment [48, 49]. Unlike E_2_, the level of P_4_ is gradually increased from pregnancy days 1–4 [50]. In the current study, ATF6α expression was up-regulated from pregnancy days 1–4, indicating that the increasing levels of P_4_ might result in the accumulation of ATF6α and that P_4_ plays a predominant role in regulating the ATF6α level in the mouse uterus during early pregnancy. Moreover, this inference was also supported by the results of hormone treatment in ovariectomized mouse. The treatment of ovariectomized mice with E_2_ inhibited the expression of ATF6α whereas a combination of E_2_ and P_4_ increased the level of ATF6α. As the early pregnancy proceeds, blastocyst attachment occurs on pregnancy day 5. We found that ATF6α was mainly present in the luminal epithelia and the stromal cells near the implanted embryo, and the overall expression level was higher in the uterus with an implantation site than in the uterus without an implantation site, suggesting that the blastocyst attachment has an inducing effect on ATF6α expression. It has been proven that the preimplantation factor (PIF) secreted from the activated blastocyst can regulate the expression of many proteins in the endometrial epithelia and the stromal cells to promote the invasion of trophoblast cells [51, 52]. In the meantime, the level of E_2_ on pregnancy day 4 is decreased on pregnancy day 5 while the high level of P_4_ is maintained [49, 50]. Prior investigation has shown that P_4_ and E_2_ play crucial roles in the establishment of endometrial receptivity and successful implantation by affecting the expression of many genes in endometrial cells [40]. Therefore, we speculate that the increased ATF6α on pregnancy days 4–5 may be involved in the establishment of endometrial receptivity and may promote trophoblast cell invasion during peri-implantation. In addition, high expression of ATF6α in the luminal epithelium and stromal cells of a uterus with an implantation site might be the result of the synergistic and inducing effect of the implanted embryo and the high level of P_4_. In other words, a cooperative relationship between embryo attachment and hormones is established to regulate ATF6α expression in the uterus during embryo implantation in vivo.

## Conclusions

In conclusion, the current findings indicate that ATF6α plays an important role in embryo implantation and the physiological process of decidualization. Moreover, ATF6α expression in the mouse uterus might be regulated by the activating effects of the embryo and the hormones progesterone and estrogen.

## Additional file

Additional file 1: (S1) Positive control. Western blot analysis of cleaved ATF6α expression in rat liver extract was performed as a positive control. **(S2)** Negative control. The normal rabbit IgG was used as the negative non-relevant IgG control. The results shown are representative of 3 independent experiments. (PDF 125 kb)

## Abbreviations

ATF: Activating transcription factor
ATF6: Activating transcription factor 6
ATF6α: The alpha isoforms of activating transcription factor 6
ATF6β: The beta isoforms of activating transcription factor 6
CREB: Cyclic adenosine monophosphate response element-binding protein
DAB: 3, 3′-diaminobenzidine
E_2_: Estrogen
HRP: Horseradish peroxidase
P_4_: Progesterone
PDZ: Primary decidualization zone
S1P: Site-1 protease
S2P: Site-2 protease
SDZ: Secondary decidual zone

## References

[1] Dimitriadis E, White CA, Jones RL, Salamonsen LA (2005). Cytokines, chemokines and growth factors in endometrium related to implantation. Hum Reprod Update.

[2] Achache H, Revel A (2006). Endometrial receptivity markers, the journey to successful embryo implantation. Hum Reprod Update.

[3] Xia H, Jin X, Cao Z, Shi T, Ma X (2014). MiR-98 is involved in rat embryo implantation by targeting Bcl-xl. Febs Letters.

[4] Paria BC, Lim H, Das SK, Reese J, Dey SK (2000). Molecular signaling in uterine receptivity for implantation. Semin Cell Dev Biol.

[5] Teles A, Zenclussen A (2014). How cells of the immune system prepare the endometrium for implantation. Semin Reprod Med.

[6] Yoshinaga K (2014). Progesterone and its downstream molecules as blastocyst implantation essential factors. Am J Reprod Immunol.

[7] Xia HF, Jin XH, Cao ZF, Hu Y, Ma X (2014). MicroRNA expression and regulation in the uterus during embryo implantation in rat. Febs Journal.

[8] Zenclussen AC, Hammerling GJ (2015). Cellular regulation of the uterine microenvironment that enables embryo implantation. Front Immunol.

[9] Haze K, Yoshida H, Yanagi H, Yura T, Mori K (1999). Mammalian transcription factor ATF6 is synthesized as a transmembrane protein and activated by proteolysis in response to endoplasmic reticulum stress. Mol Biol Cell.

[10] Zhu C, Johansen FE, Prywes R (1997). Interaction of ATF6 and serum response factor. Mol Cell Biol.

[11] Kim JW, Choi H, Jeong BC, Oh SH, Hur SW, Lee BN, Kim SH, Noer JE, Koh JT, Hwang YC (2014). Transcriptional factor ATF6 is involved in odontoblastic differentiation. J Dent Res.

[12] Chen X, Shen J, Prywes R (2002). The luminal domain of ATF6 senses endoplasmic reticulum (ER) stress and causes translocation of ATF6 from the ER to the Golgi. J Biol Chem.

[13] Adachi Y, Yamamoto K, Okada T, Yoshida H, Harada A, Mori K (2008). ATF6 is a transcription factor specializing in the regulation of quality control proteins in the endoplasmic reticulum. Cell Struct Funct.

[14] Darling NJ, Cook SJ (2014). The role of MAPK signalling pathways in the response to endoplasmic reticulum stress. Biochim Biophys Acta.

[15] Gomez JA, Tyra HM, DeZwaan-McCabe D, Olivier AK, Rutkowski DT (2014). Synthetic embryonic lethality upon deletion of the ER cochaperone p58^IPK^ and the ER stress sensor ATF6 alpha. Biochem Biophys Res Commun.

[16] Yamamoto K, Sato T, Matsui T, Sato M, Okada T, Yoshida H, Harada A, Mori K (2007). Transcriptional induction of mammalian ER quality control proteins is mediated by single or combined action of ATF6alpha and XBP1. Dev Cell.

[17] Lai E, Teodoro T, Volchuk A (2007). Endoplasmic reticulum stress: signaling the unfolded protein response. Physiology.

[18] Ruddon RW, Bedows E (1997). Assisted protein folding. J Biol Chem.

[19] Yang Y, Pei X, Jin Y, Wang Y, Zhang C (2016). The roles of endoplasmic reticulum stress response in female mammalian reproduction. Cell Tissue Res.

[20] Simmons DG, Kennedy TG (2000). Induction of glucose-regulated protein 78 in rat uterine glandular epithelium during uterine sensitization for the decidual cell reaction. Biol Reprod.

[21] Zhang K, Kaufman RJ (2008). From endoplasmic-reticulum stress to the inflammatory response. Nature.

[22] Hashida K, Kitao Y, Sudo H, Awa Y, Maeda S (2012). ATF6alpha promotes astroglial activation and neuronal survival in a chronic mouse model of parkinson’s disease. Plos One.

[23] Park SJ, Kim TS, Park CK, Lee SH, Kim JM, Lee KS, Lee IK, Park JW, Lawson MA, Lee DS (2013). HCG-induced endoplasmic reticulum stress triggers apoptosis and reduces steroidogenic enzyme expression through activating transcription factor 6 in Leydig cells of the testis. J Mol Endocrinol.

[24] Park H, Park S, Koo D, Lee S, Kong I, Ryoo J, Park Y, Chang K, Lee D (2014). Progesterone production is affected by unfolded protein response (UPR) signaling during the luteal phase in mice. Life Sci.

[25] Yung HW, Atkinson D, Campion-Smith T, Olovsson M, Charnock-Jones DS, Burton GJ (2014). Differential activation of placental unfolded protein response pathways implies heterogeneity in causation of early- and late-onset pre-eclampsia. J Pathol.

[26] Lian IA, Løset M, Mundal SB, Fenstad MH, Johnson MP, Eide IP, Bjørge L, Freed KA, Moses EK, Austgulen R (2011). Increased endoplasmic reticulum stress in decidual tissue from pregnancies complicated by fetal growth restriction with and without pre-eclampsia. Placenta.

[27] Lin P, Jin Y, Lan X, Yang Y, Chen F, Wang N, Li X, Sun Y, Wang A (2014). GRP78 expression and regulation in the mouse uterus during embryo implantation. J Mol Histol.

[28] Lan X, Jin Y, Yang Y, Lin P, Hu L, Cui C, Li Q, Li X, Wang A (2013). Expression and localization of Luman RNA and protein during mouse implantation and decidualization. Theriogenology.

[29] Lin P, Chen F, Wang N, Wang X, Li X, Zhou J, Jin Y, Wang A (2013). CREBZF expression and hormonal regulation in the mouse uterus. Reprod Biol Endocrinol.

[30] Tan J, Raja S, Davis MK, Tawfik O, Dey SK, Das SK (2002). Evidence for coordinated interaction of cyclin D3 with p21 and cdk6 in directing the development of uterine stromal cell decidualization and polyploidy during implantation. Mech Dev.

[31] Lin P, Lan X, Chen F, Yang Y, Jin Y, Wang A (2013). Reference gene selection for real-time quantitative PCR analysis of the mouse uterus in the peri-implantation period. Plos One.

[32] Vandesompele J, Preter KD, Pattyn F, Poppe B, Roy NV (2002). Accurate normalization of real-time quantitative RT-PCR data by geometric averaging of multiple internal control genes. Genome Biol.

[33] Sengupta J, Ghosh D (2014). Multi-level and multi-scale integrative approach to the understanding of human blastocyst implantation. Prog Biophys Mol Biol.

[34] Mourik MS, Macklon NS, Heijnen CJ (2008). Embryonic implantation: cytokines, adhesion molecules, and immune cells in establishing an implantation environment. J Leukoc Biol.

[35] Bai Z, Guo B, Tian X, Li D, Wang S, Cao H, Wang Q, Yue Z. Expression and regulation of Runx3 in mouse uterus during the peri-implantation period. J Mol Histol. 2013;44:519–26. 10.1007/s10735-013-9501-z 23572423

[36] Reid RJ, Heald PJ (1970). Uptake of (3H)leucine into proteins of rat uterus during early pregnancy. Biochimica Et Biophysica Acta.

[37] Li S, Chen X, Ding Y, Liu X, Wang Y, He J (2011). Expression of translationally controlled tumor protein (TCTP) in the uterus of mice of early pregnancy and its possible significance during embryo implantation. Hum Reprod.

[38] So AY, Fuente E, Walter P, Shuman M, Bernales S (2009). The unfolded protein response during prostate cancer development. Cancer Metastasis Rev.

[39] Bifulco G, Miele C, Di Jeso B, Beguinot F, Nappi C, Di Carlo C, Capuozzo S, Terrazzano G, Insabato L, Ulianich L (2012). Endoplasmic reticulum stress is activated in endometrial adenocarcinoma. Gynecologic Oncolog.

[40] Gwak H, Kim S, Dhanasekaran DN, Song YS (2016). Resveratrol triggers ER stress-mediated apoptosis by disrupting N-linked glycosylation of proteins in ovarian cancer cells. Cancer Lett.

[41] Maccarrone M, DeFelici M, Klinger FG, Battista N, Fezza F, Dainese E, Siracusa G, Finazzi-Agro A (2004). Mouse blastocysts release a lipid which activates anandamide hydrolase in intact uterus. Mol Hum Reprod.

[42] Zhang S, Lin H, Kong S, Wang S, Wang H, Wang H, Armant DR (2013). Physiological and molecular determinants of embryo implantation. Mol Asp Med.

[43] Wang H, Dey SK (2006). Roadmap to embryo implantation: clues from mouse models. Nat Rev Genet.

[44] Shao J, Li MQ, Meng YH, Chang KK, Wang Y, Zhang L, Li DJ (2013). Estrogen promotes the growth of decidual stromal cells in human early pregnancy. Mol Hum Reprod.

[45] Ozturk S, Demir R (2010). Particular functions of estrogen and progesterone in establishment of uterine receptivity and embryo implantation. Histology & Histopathology.

[46] Pawar S, Hantak AM, Bagchi IC, Bagchi MK (2014). Minireview: Steroid-regulated paracrine mechanisms controlling implantation. Mol Endocrinol.

[47] Campbell CS, Ryan KD, Schwartz NB (1976). Estrous cycles in the mouse: relative influence of continuous light and the presence of a male. Biol Reprod.

[48] Shirane A, Wada-Hiraike O, Tanikawa M, Seiki T, Hiraike H, Miyamoto Y, Sone K, Hirano M, Oishi H, Oda K (2012). Regulation of SIRT1 determines initial step of endometrial receptivity by controlling E-cadherin expression. Biochem Biophys Res Commun.

[49] Paulson RJ (2011). Hormonal induction of endometrial receptivity. Fertil Steril.

[50] Ni H, Yu XJ, Liu HJ, Lei W, Rengaraj D, Li XJ, Yang ZM (2009). Progesterone regulation of glutathione S-transferase Mu2 expression in mouse uterine luminal epithelium during preimplantation period. Fertil Steril.

[51] Stamatkin CW, Roussev RG, Stout M, Coulam CB, Triche E, Godke RA, Barnea ER (2011). Preimplantation factor negates embryo toxicity and promotes embryo development in culture. Reprod Biomed Online.

[52] Barnea ER, Kirk D, Paidas MJ (2012). Preimplantation factor (PIF) promoting role in embryo implantation: increases endometrial integrin-alpha2beta3, amphiregulin and epiregulin while reducing betacellulin expression via MAPK in decidua. Reprod Biol Endocrinol.

