# Supplementary figures and images for: Expression and regulation of ATF6α in the mouse uterus during embryo implantation
Source: Reprod Biol Endocrinol. 2016 Oct 7;14:65. doi: 10.1186/s12958-016-0199-0 (PMC5055674; doi:10.1186/s12958-016-0199-0)

Additional file 1

**S1**

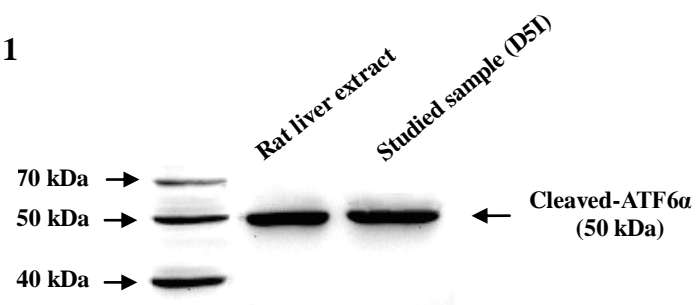

**S2**

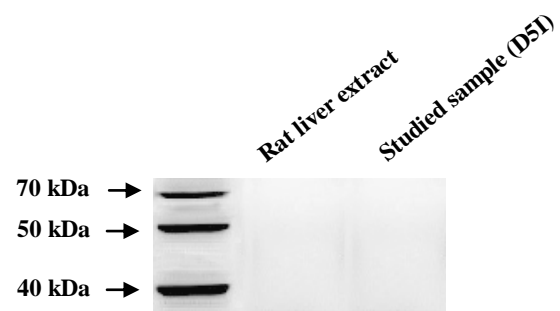

Supplement: Additional file 1: (S1) — Positive control. Western blot analysis of cleaved ATF6α expression in rat liver extract was performed as a positive control. (S2) Negative control. The normal rabbit IgG was used as the negative non-relevant IgG control. The results shown are representative of 3 independent experiments. (PDF 125 kb) [file 12958_2016_199_MOESM1_ESM.pdf]
